# Effects of a Process-Oriented Goal Setting Model on Swimmer’s Performance

**DOI:** 10.2478/v10078-012-0024-6

**Published:** 2012-05-30

**Authors:** Paulo Simões, José Vasconcelos-Raposo, António Silva, Helder M. Fernandes

**Affiliations:** 1 Research Centre in Sports, Health and Human Development (CIDESD.; 1 University of Trás-os-Montes and Alto Douro (UTAD), Portugal.

**Keywords:** goal setting, mental training, swimming, performance

## Abstract

The aim of this work was to study the impact of the implementation of a mental training program on swimmers’ chronometric performance, with national and international Portuguese swimmers, based on the goal setting model proposed by Vasconcelos-Raposo (2001). This longitudinal study comprised a sample of nine swimmers (four male and five female) aged between fourteen and twenty, with five to eleven years of competitive experience. All swimmers were submitted to an evaluation system during two years. The first season involved the implementation of the goal setting model, and the second season was only evaluation, totaling seven assessments over the two years. The main results showed a significant improvement in chronometric performance during psychological intervention, followed by a reduction in swimmers’ performance in the second season, when there was no interference from the investigators (follow-up).

## Introduction

One of the main objectives of sport psychology is to be recognized as a credible and positive influence on sports performance. To achieve this, several researchers have attempted to determine the type of goals that may have better impact on sports performance. Burton (1989) was among the first researchers to provide a study over a sports season with a sample of swimmers and concluded that the objectives should be specific, realistic and short-term, thus enhancing the process of achievement (mastery). Globally, this type of objectives tend to be associated with better outcomes regardless of the research methodology adopted (case studies, comparison between groups and / or inter-group). On an operational level, it seems clear that the ability to define objectives tends to be closely linked to the degree of effectiveness of intervention (Burton, 1989; Giannini et al., 1988; Pierce and Burton, 1998; Weinberg et al., 1993; Weinberg et al., 1994). The approaches applied have sought to define objectives for the degree of difficulty, proximity and specificity of intervention to ensure an impact, generally positive, of the tasks studied. In this regard, Burton et al. (2001) support the assumption of a positive influence on research related to setting goals, taking into account two hundred and one studies since 1984. This empirical evidence has shown that the process of goal setting was effective in improving sports performance in several tasks, and in many different contexts, including industrial and organizational (Locke and Latham, 1985, 1990; Weinberg et al., 1993). More recently, Brewer (2009) stated that, in sports context, setting goals is one of the most recognized techniques in the execution of tasks and the most frequently used by leading sports psychologists who support the Olympic athletes of the United States of America. He also shows in summary that goal setting has been used primarily to: 1) provide an atencional direction, 2) mobilize athletes’ efforts, 3) extend the persistence of the data and 4) to learn new strategies.

However, a closer examination of the studies in sport psychology shows that intervention within recreational sports are often limited in the period of intervention, the variables to be studied and in the number of subjects used, making it rather flexible and inclusive of the competitive settings with a limited degree of extrapolation. Thus, the definition of the fine line of impact between intervention and sport performance, with a reduced risk to the investigator at the level of scientific credibility, has led us often to minor evidence embodied in reality and not transferable to the current requirement’s of advanced coaching. As such, when defining objectives we consider essential the need to formulate and implement a more holistic model, which allows interpretation of the needs of various types of targets around the athlete, without ignoring quantification. In this context, Brewer and Shillinglaw (1992) highlight the need for further studies in real-world sports context, as found in this research on the application of goal setting and imagery program, which denoted the viability and effectiveness of this assumption, as long as the result is obtained from controlled conditions. In addition, there is a lack of a model that allows the integration of a mental training program (more holistic and idiographic in nature), implicitly associated with this type of intervention. Thus, in our study we have selected a model of goal setting addressed to the limitations listed above.

### The Goal Setting Model

Taking into account the development of the model proposed by Damon Burton and the maximization of its quantification in sports context, Vasconcelos-Raposo (2001) proposed a model of goal setting specific to sports reality and conceived based on the competition process, thereby seeking to achieve its purpose according to what are the habitual practice of coaches when planning the training session in preparing athletes for competition. This was accomplished by taking into consideration the following aspects: 1) the qualitative goals associated with the coach′s intervention translated into the basic technical elements that integrate the competitive process, and 2) quantitative competitive goals that sustain and direct the conduct of the athlete and coach.

The model proposed intends to provide additional information to the coach allowing him to make the necessary adjustments in his training schedule, namely at the level of intensity and loads, and consequently adjusting the swimmers’ competitive mental and physical plan for the competition.

This model focuses in technical and performance elements, considered key to analyze the efficiency of the swimmer during the competition. The main goal is to develop the athlete′s self-sufficiency capacities to make decisions, during the competition (depending on the distances), regarding the energetic resources they perceive available and consequently decide to intensify (or not) their effort and at what distance from the finish they should act. Another aspect considered relevant in the model is that both coach and athlete, once the competition is over, based on the objective information gathered, are able to discuss and adjust the following training cycle sessions in order to overcome the deficiencies identified during the performance. The variables used in the adopted goal setting model are: “start-time”, number of swimming cycles, “time-turns” which is subdivided into two moments, time-in and time-out, number of swim cycles during the second 50 meters, for example, and the finish-time. Based on previous discussions between coach and athlete the latter should be able to evaluate his/her capacity to take risks in spending an extra effort to better the overall time pre-defined for the competition in question.

The implementation of Vasconcelos-Raposo (2001) proposed model does not preclude the relevance of each type of goals as they are commonly defined in term of short versus long-term goals and how they need to be articulated with each other. Short-term goals are translated and workout throughout the training sessions according to the coach’s planning to improve the physical conditioning, technical and mental skills needed to implement the swimming strategy designed in order to attain certain final time goals. According to Weinberg et al. (1994) this type of goals tends to produce a larger effect on the athlete’s competitive performance. Nevertheless, and according to Vasconcelos-Raposo (2001), the long-term goals are essential to keep the swimmers focused on their career plan, serve as benchmarks and give direction and persistence to the athlete (Weinberg, 2009). On an operational level, the integration of these multiple objectives emerge as a method to drive the swimmers/athletes to a better understanding of the factors involved in the achieving better results as a natural consequence of the individual dedication, concentration and effort put into training sessions. This educational context tends to enable a higher commitment and motivation to the coach’s plans. In order to achieve this, and most importantly in our perspective, goals must be constantly redefined in every moment of assessment and in accordance with the swimmer’s mental toughness (Loehr, 1986) and performance profile. With the evaluation system, we intend to provide a functional interpretation of events and involve the athlete and coach in the process of maximizing performance.

It is also essential, as a basic concept, to understand that training and competition are two different units of analysis, although contiguous, in that: 1) the training process is a requirement for the competition and 2) the competition is a product of the training, which, in turn, decomposes into multiple components and requires various types of energetic demands. Thus, it is proposed that the competition is seen as a process of management of energy resources (physiological process) directly under the control of the athlete (mental process) otherwise, the development of training plans according to energy solicitation would be pointless. As such, without a thorough and multidisciplinary study it is not possible for either the coach or the athlete to develop the ability to establish a causal relationship between work and achievements.

However, Brewer (2009) warns that setting goals only makes sense if you create an action plan to achieve these objectives, this serving as a process of sequencing attention (concentration) to the various hierarchic objectives settled (Weinberg, 2009). In this case (study of multiple cases), for this to happen, there must be a facilitator and responsible process for the internalization of the model – the mental training program.

### The Mental Training Program (MTP)

Several studies have attempted to demonstrate the positive impact of intervention based on goal setting. However, other techniques in sport psychology have often been used and developed in competition setting (relaxation, imagery, self-talk, atencional training). Our proposal tries to integrate them in a broad plan of intervention using the goal setting model as a guideline.

Evans, Jones and Mullen (2004) in this regard, argued that future research should seek to explore the relationship between the definition of objectives, cognitive orientation and imagery. Adding to this perspective, there should be a study to establish inferences causes between imagery and performance, using for this purpose, a systematic analysis of the outcome. Vasconcelos-Raposo’s (2001) model tries to respond to some of these suggestions, assuming a systematic application of various techniques of mental and physical order for the effective internalization of the objectives through a mental simulation of the competitive event, which encompasses other resolution strategies and psychological control, according to the causes identified. As such, several intervention techniques, like simulation imagery, atencional control, analysis of education through video, control of the negativity and kind of thoughts, integration of triggers, increased sensitivity to water management and effort are some of the possibilities of the operationalization of the MTP. This study enabled the understanding of the model of goal setting and its relationship with the proposed methodologies for technical training, as well as effort management in the swimming event.

Considering that, this model shows its greatest benefit in the individualization of training with swimmers (idiographic approach), common strategies are not overlooked on all elements of the sample, although these are tailored to the needs of each individual according to the interpretation data brought out from evaluation. Therefore, throughout the intervention process inherent in the present study, some of these strategies were the responsibility of the coach and our intervention regarded merely guidance, when necessary.

In the current research, therefore, we assessed the impact of the implementation of a goal setting model in its full and integrated form in the coach’s planning. In this sense, we established as our main aim to determine the effects of this model as proposed by Vasconcelos-Raposo (2001) on national and international swimmers’ chronometric performance.

## Methods

This study is a longitudinal design follows a single-subject study with a multiple baseline design (Landin and Hebert, 1999), which allows us to combine aspects of more detailed empirical analysis (idiographic approach) with some of the assumptions associated with the positivist perspective, converting into a mixed research methodology.

### Sample

The participants of the study were nine Portuguese swimmers, who were followed during two competitive seasons. Of these, four were male and five female, aged between fourteen and twenty years (*M* = 17.44, *SD* = 1.42) at the initial moment of collecting data. All swimmers had five to eleven years of competitive experience (*M* = 7.44 years), regularly participating in national and international events. These swimmers belonged to the same sports club and were under the same coach’s leadership and training.

### Procedures

The chronometric records were obtained in a competition state with official referees of the Portuguese Swimming Federation, according to guidelines adopted by the European Swimming League (ESL). Only the first assess took place in the context of training. During the swimming event video recordings were also used to verify the chronometric timings (partial and total performance) such as, start time (15 m), split time (50 m), time of entry for the turn (5 m from the wall), time out of the turn (10 m from the wall), finishing time (last 10 m) and swimming time (total time minus the start time, finishing time, and turns). This protocol complied with the proposals of Smith, Norris and Hogg (2002).

Throughout the process of data collection, all participants were subjected to seven moments of assessments at the same time in each season (see table 1). With the exception of the first moment (Time 1 – T1), held in a state of training, all the rest occurred in a situation of official competition – national championship.

In the first season, evaluation took place at the same time as the period of the application of the model of goal setting, that is, in a weekly basis for a period of thirty minutes per session, followed by physical training. As for the second season, it did not contemplate any form of intervention but merely an evaluative period without intervention (follow-up).

Thus, our approach allows us two forms of analysis. A longitudinal analysis from T1 to T7 (analysis of developing inter-moments) and cross analysis between T2–T5, T3–T6 and T4–T7 (comparative analysis of inter-season). In this context, it becomes possible to evaluate the retention values and at the same time compare the two seasons. This way we were able to discuss and give feedback about the variability of the athlete’s performance and make swimmers aware of their moments of fitness, ability and also their swimming skills. We could also create short-term expectations among the swimmers and the coach breaking down the season’s goals.

As to initial procedures, we contacted the technical director and coach, to inform them of the purposes of our study in order to obtain their approval and full support. In the first meeting, the coach introduced the experimenter (mental preparation coach) to the swimming team and informed them that the study was focused on mental preparation in two situations, training and competition. This initial process helped the experimenter’s integration - the coach showed support and interest in this type of work, which was also shared by the swimmers.

At the beginning of the first season, the experimenter met several times with the swimmers in order to standardize the terminology and interpretation of the psychological and dimensional techniques implied in the training program and inherent to the mental evaluation. Subsequently, the first chronometric assessment in situation of training (T1) was completed, which provided the swimmer, the coach and the experimenter with a functional understanding of his/her performance, making it possible to relate the data of chronometric swimming with the psychological domain.

Following, regarding the planning of training sessions based on the postulates of the goal setting model, were defined season performance times with the swimmers and the coach, which were used as referential to the adaption of individualized strategies inherent to the mental training program (MTP). The following table aims to present in a summary form, the implementation of the mental training program adopted, and taking into account the guidelines proposed by Goginsky and Collins (1996).

After each moment of evaluation in the intervention season (T2 to T4), the experimenter along with the swimmers and the coach constantly redefined the initial goals settled projecting resolution exercises to improve performance and to be included in future planning. This feedback allowed a balance between long term and short-term goals.

During the time of follow-up, the swimmers were not subjected to any kind of mental training. However, the coach without the intervention of the experimenter continued to provide them with technical and timing information in the same form as the one given in the first season.

### Statistical analysis

After inserting performance chronometric data on a spreadsheet, we proceeded to the standardization of the data of each swimmer by moments of evaluation taking into account the equivalence according to mean and standard deviation of each sport season. To reach this end we applied the following formula:

$$Z=\frac{X-\mu }{\sigma }$$

With this equation, we obtain a *z-score* standardized according to the division of the result of the difference in value to normalize (*X*) and mean (*μ*) by its standard deviation (*σ*) of the range of values. After the estimation of *z-score*, the calculation of central tendency (mean) and confidence intervals (CI) of 95% was carried out for each evaluation moment using the SPSS 16. In order to determine the degree of variation of mean values in time, repeated measures ANOVA were performed with a significance level set at 5% (*p* < .05).

## Results

The performance time in seconds (s) of each swimmer is given in table 3, taking into account the different moments of evaluation. Again, the result of the T1 was obtained in competitive situation simulated in the context of training, while the other records were obtained in national swimming events in official competition as can been observed in following table.

According to a longitudinal perspective of this research we have collected data of chronometric performance of all the swimmers for all the moments of evaluation, with the exception of swimmer number 9 (400 medley) in moment T7, who did not attend the last race of the follow-up due to dropout. Based on the identification of best individual performance, T4 matched the best personal times of swimmers numbers 3, 4 and 5. On T5, swimmer number 7 reached his best personal record, while the values of T3 and T5 were equivalent to the best personal time of swimmer number 8.

**Figure 1:**
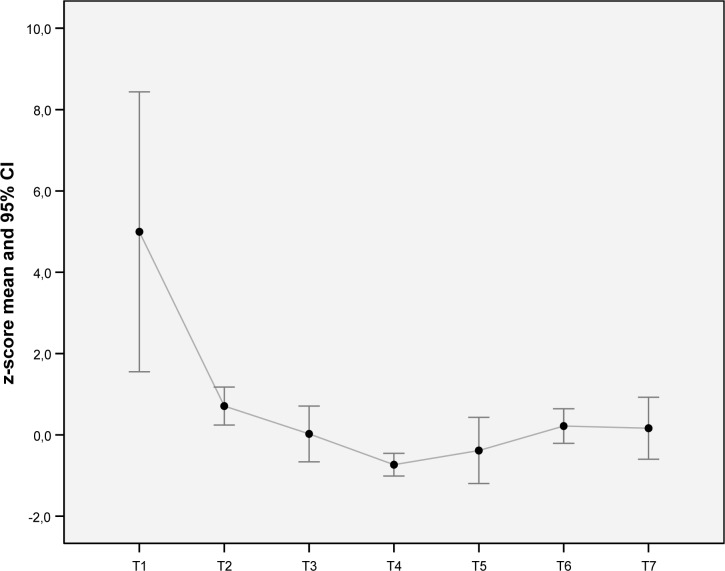
Mean and 95% confidence interval of z-score per moment of evaluation

Figure 1 shows the mean value and its confidence interval 95% of the *z-score* for each time of evaluation.

Data reveals a general reduction in swimming time (better performance) between T2 and T4, reaching by the end of intervention season the lowest mean *z-score* followed by a smaller amplitude (95% CI), which suggests a positive increase of performance as well a greater uniformity of swimmers’ sports performance. After the end of the period of intervention (T4), it is denoted a decline of the outcome that leads to an increase in the mean *z-score*, which tends to stabilize at the end of the period of follow-up (T6 and T7), although values of mean and 95% CI are higher than T4.

The overall of the swimming data was examined in a longitudinal perspective by repeated measures analysis of variance. We verified a positive and significant development of performance throughout the intervention season, *F*(5, 35) = 3.22, *p* < .02. The analysis of contrasts between measures (within-subjects), taking as reference the initial evaluation in competitive settings (T2), revealed significant differences for the pairs T4-T2, *F*(1, 7) = 100.07, *p* < .001, and T5-T2, *F*(1, 7) = 8.39, *p* < .05, although the variability explained is much higher for the first case (η_p_^2^ = .935 and η_p_^2^ = .545, respectively). For post-hoc comparisons between all time points with the Bonferroni method, only significant differences were noted between T2 and T4 (*p* < .001), showing a significant improvement in performance along the profile during intervention season.

## Discussion

The purpose of this study was to analyze the effect of the goal setting model focused on the swimmers’ chronometric performance. Primarily, given the specificity of the data collected for different distances and styles, there was the needed to standardize scores. Secondly, this normalization of the time achieved between different events (*z-score*) allowed us to establish a connection between all swimmers in different moments of assessment and explain the differences in performance of the whole group of swimmers along the two seasons. Thus, it was possible to identify a significant reduction in the chronometric outcome during the intervention period (T2 *vs* T4, *p* = .009).

It would be expected that, even without the intervention, swimmers would improve due to factors of maturity and accumulated practice. But when analyzing other important data regarding the comparison of the intervention and the follow-up, we can better understand its importance. There was a significant decrease of performance in the year that immediately followed the application of the model of goal setting (follow-up). This phenomenon is reinforced when we compare the results obtained at the end of each season. At the end of the intervention period the swimmers’ benefits were significantly better than at the end of the following year (T4 *vs* T7, *p* = .045). In this context, other results tend to back up this argument. There were no significant differences between early intervention and the end of follow-up (T2 *vs* T7, *p* = .187), it seems clear that swimmers’ outcome by the end of follow-up went back to those obtained before intervention, highlighting the temporality of learning in the absence of stimulation. Therefore, it seems that there was a quantitative setback of chronometric performance associated with the lack of mental training practice and goal setting feedback, therefore, this fact partially justifies the relevance and importance of the application of this model.

When analyzing the transition between seasons, it seems clear that this decline is not sudden and abrupt in time. The effects of intervention are verified in the closest evaluation moment after intervention and decline progressively after. According to the statistic data obtained, performance by the end of intervention and the beginning of follow-up is not significantly different (T4 to T5, *p* = .325). However, during the second season (follow-up) we can verify discrepancies between the longitudinal chronometric pairs of assessments. There are significant differences between T4 and T6 (Δz = 1.00, *p* = .001) and between T4 and T7 (Δz = .90, *p* = .045) indicating that performance outcome declined when compared with the end of first season. Although there are several possible explanations for this evidence, Johnson, Ostrow, Perna and Etzel (1997) help us to justify the lack of significant differences in the transition between the end of the first season and the beginning of follow-up (T4 *vs* T5) close to intervention. According to these authors, there may have been an additional justification for maintaining good results. Concerning our sample, the season’s first official review (T5) was the Clubs National League and the team’s objectives may have lead to a positive effect on personal goals since the teams’ expectations were high (holding the title of female national champions and changing to an upper rank for the boys). Comparing the moments in the middle of each season we also found that there were no differences, leading to complete this evolutionary framework. There was a positive and significant swimming profile along the first season, and a significantly regressive follow-up season in the absence of this project. The comparison of these moments lead us to understand the consequences associated with quantitative research, as well as to better understand the implications of the methodological process adopted to achieve it.

In summary, in the year of intervention the swimmers remained defiantly close to their targets, outcome of their personal investment (time and energy) therefore achieving results within or slightly below the reference system designed by them (Chelladurai, 1991) and the model in question. Some possible explanations in psychological literature may explain part of these phenomena and this clear pattern of behavior. The proximity of the swimming events to the proposed object leads us to comprehend that the intervention changed the way the swimmers interpret, feel and commit to the process, making them more persistent (Weinberg, 2009), more aware of their swimming ability and capable to understand the processes under their control. This idea is backed up by the evidence of its regression when we analyzed the data collected from follow-up. During that year, no mental training was held along with the performance, with what was expected or what came to occur (lower functional equivalence). There was also a greater difference between the chronometric mental event and what would be expected and what came to occur. As proposed by Vasconcelos-Raposo (2001), a swimming event consists of a physical, technical and an energetic management under a cognitive and emotional control that should be synchronized, in this case, to the process of control lowered in the second season.

According to Bagozzi (1992) perspective, swimmers probably created a greater commitment to the various levels of goals established, developing higher levels of effort and approaching their performance to the objectives defined within the project. We believe that with intervention this group of swimmers was interested in learning the dynamics of the process, how to detect specific causes and reset new operational goals.

They sought to organize and control their emotions and actions based on the correct interpretation of the causes that led them to the results obtained, according to ability, personal effort and difficulty of the task (Weiner, 1986). This way we expected to increase the internal locus of control in the diagnosis of the causes of error and how to use them for their own advantage (Vasconcelos-Raposo, 2001). In this sense, we lead athletes to better interpret the causes and learn how to use this information for their own benefit. These facts mediate expectations, emotions, motivation and behavior (DeCharms, 1976). We were always concerned during the analysis of the data that the controllability of a closed task such as swimming was the responsibility of all of those involved, the technical team and the swimmer. This way we would help stabilize or improve the results throughout the swimming season (Biddle, 1993). We believe that the way swimmers interpreted or were led to interpret every swimming event positively affected the subsequent behavior and outcome.

We can also state that the way the swimmers decided to define their objectives within our framework was influential in the results expected and achieved when compared to the results obtained with the second season in the absence of intervention / stimulation. These data confirm the importance of the chosen techniques in the context of competitive sport essentially when integrated with the model of intervention. Its absence contributes to a possible lack of motivation and less commitment with goals, as well as possible lower cognitive and emotional self-control. The absence of information from assessments during the follow-up made the swimmers less aware and less objective regarding behavior constructing more subjective levels of action. For Webb and Sheeran (2005), regarding this matter, the study of objectives when proposed should be in a context of self-fulfillment in order to better understand its importance, as our proposal suggests. Only in this way will these reveal their interest and impact as we aimed to in this study.

Considering the fact that the swimmers came to set more realistic goals throughout the intervention period (taught to be defined) created a greater degree of compromise with training (attendance and intensity). This idea is reinforced by interviews held with the coach about the comparison between seasons. The coach’s planning was influenced by the quantity and quality of information available. For this matter and in his opinion, the goal setting model lead him to take better decisions in managing the load of training and the negotiation / collaborative process in establishing goals and exercises (Weinberg et al., 1993).

On the contrary, in the second year in the absence of the evaluation protocol the objectives became more ambiguous and therefore more difficult to interpret and harder for the coach’s decision-making. Agreeing with the coach’s opinion and with those of Burton et al. (2001), evaluation is one of the most critical steps in applying a plan in defining goals. Athletes only benefited from the process of setting expectations (as proposed, multi-objective) when they were allowed to evaluate and understand the development of their performance. Duda (1993) summarizes effectively the influence of the theory of goal setting, stating that goals not only provided a directional focus but also improved decision-making and influenced outcome. He adds that the swimmers were not only passive elements that respond to reinforcements or punishments, but had an active role in the interpretation of the experiments and the construction of future expectations. We believe that the program of mental training based on a broad framework such as the goal setting model proposed by Vasconcelos-Raposo (2001), in a general form, created greater accuracy and awareness about themselves as well as the way they interpreted the psychological events that occurred and how these had a positive influence in their performance.

For many authors (Cumming and Ste-Marie, 2001; Feltz and Landers, 1983; Hall, 2001; Harwood et al., 2003) imagery tends to be an important strategy for sports success, facilitating performance through cognitive repetition, i.e., through mental training. The mental swimming plan created an opportunity to anticipate and practice pre-competitive states, seeking to reduce the probable lack of self-control.

The combination of techniques and strategies adopted in mental training seem to be fully inclusive in the model of goal setting and are an advantage in competitive sports.

## Conclusion

The goal setting model proposed by Vasconcelos-Raposo (2001) and the chronometric data collected over two seasons, allowed us to verify a significant and positive growth in group performance. The intervention (first season) seemed to be associated with the improvement of the group’s sports performance. On the other hand, in the second season, without any psychological intervention, the swimmers tended to increase their chronometric record, showing a drop in performance compared to their timing obtained at the final evaluation of the first season.

In addition, goal setting can be considered an overall context in which mental training can be integrated. We think it is more important for sport psychology to define the best psychological strategies in a single-subject perspective to cope with evaluated causes in sports situations. A general base line, as this model, will sustain the selection of cognitive and emotional techniques to influence sports behavior and a psychological profile (Weinberg et al., 2001).

Thus, it is important to understand that part of the empirical evidence obtained in this study results in terms of psychosocial dynamics which was collected in parallel with this research project and will be examined in future articles. In this context and in order to contribute to the advancement of knowledge in the field of sport psychology, it is important to examine the theoretical relevance of scientific content associated with psychological profiles of the group in sports performance (Jackson et al., 2001). So, it becomes important to present the swimmers’ psychological profiles of this studied deriving from this intervention, in order to better understand their participation and synchronization with the performance outcome as presented here.

However, some methodological limitations were evident that should be reduced or eliminated in future research on this topic. Although the model emphasizes an idiographic approach this research could benefit from a larger size of the sample, as well as the existence of a control group, which would allow an analysis of contrasts between chronometric performances of the subjects. Another limitation concerns the restricted presentation of the range of theoretical and empirical framework of the model (Vasconcelos-Raposo, 2001), which has not been widely portrayed in this research. In this sense, and being a bio-psychosocial approach to the athlete, future research should seek to relate the psychological dimension in mediating the degree of goal setting effectiveness on performance. Thus, the methodological limitations set out above suggest some caution in the interpretation and generalization of the results, it is therefore necessary, an increase of future research on the implementation and operationalization of this type of studies focused on the process of goal setting, both in individual and in team sports.

In sum, this research is effective in enhancing swimmer’s performance confirming the significance of goal setting in sports, corroborating previous studies (Burton, 1989; Giannini et al., 1988; Pierce and Burton, 1998; Salmela, 1989; Weinberg et al., 1993, 1994). Finally, the longitudinal perspective given of the intervention season followed by the assessment of a season without any interference from the investigators (follow-up), as well as, the focus on process-oriented approach are assumptions worthy of development, both in the scope of sport psychology and in future research of this nature.

## Figures and Tables

**Table 1:** Moments of evaluation during the two seasons

	October	December	March	July
Season 1 – Intervention	T1	T2	T3	T4
Season 2 – Follow-up		T5	T6	T7

**Table 2:** Mental Program Training

Task	Protocol / instruments
Time of session (Mental Training)	30 minutes /weekly
Instructions	Verbal description according to coach’s terminology
Mental Training (MT)	Progressive construction of the task according to partial chronometric data regarding final time and the goals defined progressive simulation from partial times to full construction of mental race
Physical Practice (PP)	Coping strategies were learned and transferred into swimming practice according to causes of mistake
Number of MT sessions	1 swimming season (36 sessions)
Similarity of MT with idealize performance	100%
Additional technical support	Audiovisual data of swimming events in competitive settings Video analysis with a 5 set system of cameras in competitive event according to ESL protocol

**Table 3:** Chronometric data from swimming event along the two seasons

Swimmer	Intervention Season				Follow-up Season		
	T1	T2	T3	T4	T5	T6	T7
1 (200 Backstroke)	153.68	147.53	152.76	144.28	146.24	146.63	144.46
2 (200 Butterfly)	146.84	149.53	139.29	141.41	143.57	139.53	140.52
3 (100 Breast)	83.85	77.21	77.96	74.98	75.79	77.24	79.69
4 (200 Freestyle)	141.6	135.64	133.61	131.1	131.1	133.13	133.61
5 (200 Butterfly)	144.16	129.89	126.41	126.17	129.82	129.11	126.47
6 (400 Freestyle)	293.1	268.52	271.59	265.38	265.25	274.55	277.49
7 (100 Freestyle)	56.21	55.87	54.47	54.92	52.74	53.69	53.49
8 (100 Breast)	76.82	69.73	68.62	68.65	67.02	67.74	68.24
9 (400 Medley)	309.93	301.18	289.76	288.35	245.52	258.55	—

*It is presented for each swimmer the chronometric data in total seconds by moment and style swam*.
